# Self-Expanding Anchors for Stabilizing Percutaneously Implanted Microdevices in Biological Tissues

**DOI:** 10.3390/mi12040404

**Published:** 2021-04-06

**Authors:** Sharath Bhagavatula, Devon Thompson, Christine Dominas, Irfanullah Haider, Oliver Jonas

**Affiliations:** Department of Radiology, Brigham and Women’s Hospital, Harvard Medical School, 75 Francis Street, Boston, MA 02115, USA; devon.thompson96@gmail.com (D.T.); cdominas@bwh.harvard.edu (C.D.); ihaider@bwh.harvard.edu (I.H.)

**Keywords:** implantable microdevice, device anchoring, interventional radiology, self-expanding anchor

## Abstract

Percutaneously implanted miniaturized devices such as fiducial markers, miniaturized sensors, and drug delivery devices have an important and expanding role in diagnosing and treating a variety of diseases. However, there is a need to develop and evaluate anchoring methods to ensure that these microdevices remain secure without dislodgement, as even minimal migration within tissues could result in loss of microdevice functionality or clinical complications. Here we describe two anchoring methods made from biocompatible materials: (1) a self-expanding nitinol mesh anchor and (2) self-expanding hydrogel particles contained within pliable netting. We integrate these anchors into existing drug-screening microdevices and experimentally measure forces required to dislodge them from varying tissues. We report similar dislodgement forces of 738 ± 37, 707 ± 40, 688 ± 29, and 520 ± 28 mN for nitinol-anchored microdevices, and 735 ± 98, 702 ± 46, 457 ± 47, and 459 ± 39 mN for hydrogel-anchored microdevices in liver, kidney, fat, and muscle tissues, respectively—significantly higher compared with 13 ± 2, 15 ± 3, 15 ± 2, and 15 ± 3 mN for non-anchored microdevices (*p* < 0.001 in all tissues). The anchoring methods increased resistance to dislodgement by a factor of 30–50× in all tissues, did not increase the required needle gauge for insertion, and were compatible with percutaneous implantation and removal. These results indicate that anchoring significantly improves microdevice stability and should reduce migration risk in a variety of biological tissues.

## 1. Introduction

There has been increasing emergence of implantable device technologies that can be directly inserted into patients’ tissues (in vivo) to carry out specific functions. Such technologies include fiducial markers and brachytherapy seeds for radiation treatment [1,2], biopsy markers for tumor localization, and emerging preclinical and early clinical devices for precision drug delivery, disease monitoring, and early detection [3,4,5,6]. As these devices interact directly with live tissues in their native environment, they can greatly improve diagnostic and therapeutic clinical capabilities. In particular, one recently developed implantable microdevice (IMD) shows promise for evaluating multi-drug response in cancer patients [7]. This IMD is implanted directly into a tumor and delivers micro-doses of up to 20 distinct drugs into separate microscopic regions of the tumor. After ~1–3 days to allow each drug to interact with the tissue, the microdevice and adjacent tissue are removed and evaluated histologically to determine the efficacy of each drug and identify the optimal systemic treatment. This allows numerous drugs to be tested immediately without exposing patients to prolonged trials of toxic and potentially ineffective systemic chemotherapy trials. This has been shown in several pre-clinical models to be capable of optimizing systemic treatment strategies, and early clinical trials have also been promising [7,8,9,10].

One important feature of this and other implantable devices is the ability to fit coaxially within a thin needle (17-gauge or smaller). This allows them to be placed into specific tissue sites using a minimally invasive percutaneous technique, wherein the needle is guided to the target under image guidance and the device is deployed. This can be performed as a short (<1 h) outpatient procedure without requiring large surgical incisions. The ability to implant devices in this manner using small-gauge needles, rather than a comparatively much higher risk and higher morbidity inpatient surgical implantation, has enabled biopsy markers, fiducial markers, and brachytherapy seeds to become standard of care tools widely used in cancer patients. It has also allowed enrollment of a greater number of patients into early clinical trials evaluating the feasibility of the drug-screening IMD [11,12,13], and should allow more widespread adoption of this technology in a clinical setting. In addition, a recently developed minimally invasive retrieval method allows non-surgical removal of the IMD [14]. With these advances, the IMD and similar implanted devices can be placed and removed (if appropriate) in a relatively safe manner that minimizes procedural risk and recovery time.

One limitation of these implantable devices is their propensity to migrate within tissues, either locally within the target site, or distally into other organs if they embolize through blood vessels. Such migration or embolization can lead to loss of functionality, bleeding, non-target organ injury, or other serious complications. Migration rates of up to 36% have been reported with implanted fiducial markers and brachytherapy seeds, resulting in loss of efficacy and occasionally even life-threatening complications, particularly when they embolize distally into the lungs and heart [15,16,17]. Biopsy marker migration rates are also high, with one recent study reporting a 13% migration rate in breast tissues [18]. Although early pre-clinical and clinical studies evaluating the drug-screening IMD have been overall promising, they have also identified migration to be a potential barrier to clinical translation. Because IMD functionality relies on sub-millimeter precision delivery of drug into adjacent tissue, it is essential that the implant remain fixed within the tissue after delivery. We have observed migration during pre-clinical trials and in early clinical trials, predominantly along the long (central) axis of the cylindrical device, which has led to both loss of functionality and inability to retrieve IMDs. Therefore, there is a need to develop and test methods to secure such percutaneously inserted devices in place after delivery into tissues.

Here we describe two anchoring methods that are integrated into our drug-screening IMD to prevent migration. In the first method, a nitinol mesh anchor is attached to one end of the IMD. The anchor collapses to a narrow diameter while inside the delivery needle, and expands mechanically upon release into the target tissue. In the second method, a novel approach using expandable hydrogels is presented. Expandable hydrogel particles are contained within pliable mesh netting attached to the IMD. The particles expand rapidly upon contact with water in tissues, leading to an enlarged gel mass that is contained within the mesh netting. Importantly, both methods utilize biocompatible, commercially available, FDA-approved technologies that are currently used as breast biopsy markers (placed percutaneously to mark a biopsy site and enabling subsequent surgical tumor localization and resection if pathology confirms malignancy), thus enabling potentially broad use without the additional regulatory hurdles associated with unapproved materials [19,20]. These were originally developed in response to high migration rates observed with first-generation non-self-expandable biopsy markers [18], and early tests have found them to reduce migration risk in breast cancer patients [21]. However, use of such materials as generalizable anchors to stabilize other implantable microdevices has not been described, and direct quantitative force testing to evaluate and compare their stabilizing effects in a variety of tissues have not been performed. Such studies are needed to assess the feasibility of these anchoring methods and inform further clinical translation.

In this study, we integrate nitinol- and hydrogel-based self-expanding anchors into our custom drug-screening IMD, quantify the relative stability provided with each anchoring method, and compare the stability of anchored versus non-anchored IMDs. We measure performance in a variety of commonly encountered biological tissues (fat, muscle, kidney, and liver) with varying physical properties, in which similar microdevices may be utilized in the future. Finally, we demonstrate that these anchoring methods are compatible with percutaneous implantation and retrieval. The anchoring methods described here could be integrated into any percutaneously implanted microdevice. Therefore, the results from this study should enable widespread adoption of self-expanding anchors into a variety of implanted microdevices, improving their efficacy and minimizing complication risks.

## 2. Materials and Methods

### 2.1. Implantable Microdevice (IMD) Assembly

#### 2.1.1. Non-Anchored IMD

The drug-screening IMD was machined as previously described [7,14]. Solidworks (Dassault Systems, Inc., Waltham, MA, USA) and Mastercam (CNC Software, Inc., Tolland, CT, USA) were used to develop computer-aided design and manufacture inputs. A CNC milling machine (TN5-V8-TC8, MDA Precision, Inc., Morgan Hill, CA, USA) was used to fabricate the IMD from stock material. The main IMD body, designed to sit within a tumor and deliver drug into adjacent tissue, is made from biocompatible polyetherketoneketone (PEKK) plastic. It is a 7 mm long and 750 um diameter cylinder. Its cylindrical shape allows it to fit coaxially into a 17-gauge needle for minimally invasive delivery. It has 20 discrete drug release reservoirs, each 200 um in diameter and 200 um deep. Reservoirs can be loaded with various drugs during pre-clinical and clinical trials. In this study, as we did not evaluate drug response, these reservoirs were left empty. 

At its proximal end, the microdevice body is attached to a 0.008 inchdiameter, 10 cm long nitinol wire (Malin Co., Cleveland, OH, USA). After microdevice delivery, this wire is left externalized and enables minimally invasive retrieval of the microdevice, as previously described [14]. In this study, traction on this wire is used produce tensile force on the microdevice to test stability, as described below.

#### 2.1.2. Self-Expanding Nitinol-Anchored IMD

**Figure 1:**
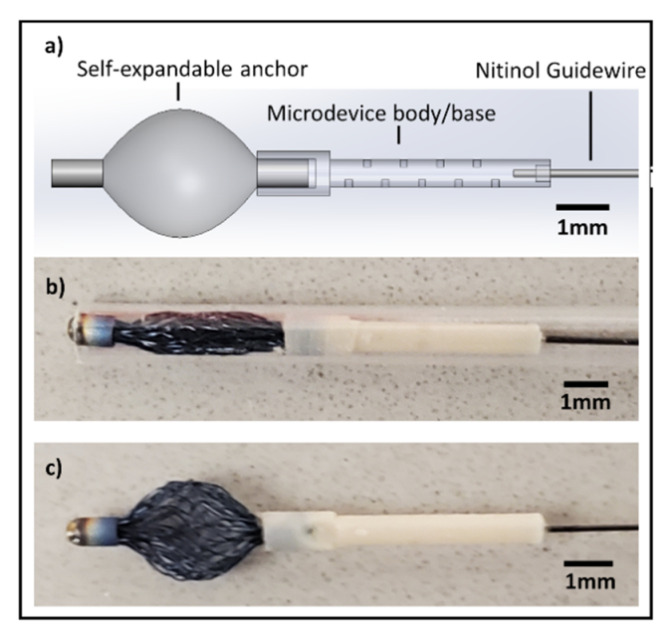
Drug screening microdevice with nitinol anchor attachment. (**a**) Schematic demonstrates the microdevice, which consists of a body with tiny drug reservoirs for precision drug release, a guidewire that enables subsequent localization and retrieval of the microdevice, and a self-expandable anchor that keeps the device secure in tissues. (**b**) Nitinol anchor is shown in collapsed form, which can be loaded into a 17-gauge needle. (**c**) After deployment, the anchor spontaneously expands, increasing the tissue contact surface area for enhanced mechanical stability.

The nitinol-anchored IMD is shown in Figure 1. The body of this IMD is machined similar to above, except its distal end is attached (by press fit and epoxy) to a nitinol self-expandable anchor. The nitinol anchor used in our study is currently FDA approved as a breast biopsy marker and compatible with long-term implantation in patients who have had a breast biopsy (Tumark Vision, Hologic, Inc., Marlborough, MA, USA) [19]. It is composed of linear nitinol fibers fastened together with a nitinol cap on either end (Figure 1b,c). In collapsed form (Figure 1b), the nitinol fibers are pushed together into a cylindrical configuration, measuring 1.2 mm in diameter and 7 mm in length, and it fits coaxially within a 17-gauge needle. Upon release from the needle, the nitinol fibers immediately expand mechanically into their natural bowed configuration, causing the anchor to balloon outward to form a spherical configuration with a significantly enlarged 3.5 mm diameter (Figure 1c). A nitinol wire is again attached to the proximal end of the IMD in an identical fashion to above. 

#### 2.1.3. Self-Expanding Hydrogel-Anchored IMD

**Figure 2:**
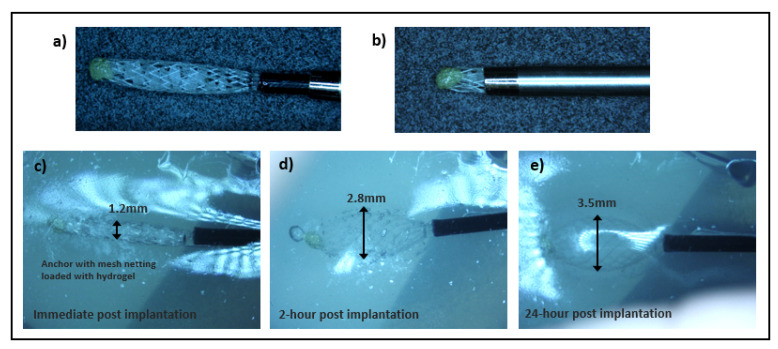
Hydrogel-anchored microdevice. The anchor consists of hydrogel water-absorbable particles contained within bioabsorbable mesh netting (**a**), which in its collapsed form can be loaded into a 17G needle for percutaneous implantation (**b**). After deployment into phantom (**c**), the hydrogel gradually expands by absorbing water (**d**). Maximal expansion is noted by 24 h after deployment (**e**).

The body and guidewire of the hydrogel-anchored IMD is identical to the nitinol-anchored IMD described above. However, its distal end is press fit and epoxied to a bioabsorbable glycoprene mesh netting that is also FDA approved and commercially available as a breast biopsy marker (SecurMarkTM, Hologic, Inc., Marlborough, MA, USA) [20]. This netting is loaded with expandable hydrogel particles of two distinct types: larger high absorption particles with 50–800 um pre-expansion diameter and 500x wt/wt absorption in deionized water (H-600, JRM Chemical, Inc., Cleveland, OH, USA), and smaller lower-absorption particles with 50–300 um pre-expansion diameter and 300x wt/wt absorption in deionized water (H-200, JRM Chemical, Inc.). The overall anchor consisting of the hydrogel particles within the netting (Figure 2a) measures approximately 7 mm in length and 1.2 mm in diameter in collapsed form, similar to the collapsed nitinol anchor. The entire IMD assembly, including the body and anchor, fits within a 17-gauge needle for minimally invasive deployment (Figure 2b). Upon deployment, the hydrogel absorbs water from the surrounding tissue and expands locally within the netting to increase contact surface area and stability (Figure 2c–e).

### 2.2. Minimally Invasive Implantation

Each of the IMDs described above were implanted into gelatin phantom (Aquaflex gel pad, Parker Laboratories, Inc., Fairfield, NJ, USA), ex vivo porcine fat, muscle, kidney, and liver tissues in a percutaneous manner, similar to methods used for fiducial and biopsy marker delivery [1,22] (Supplementary Figure S1). They were pre-loaded into a 17-gauge introducer needle (Merit Medical Systems, Inc., Rockland, MA, USA). The needle was advanced at least 2 cm into the phantom/tissue. For the tissues, the needle was advanced under ultrasound guidance, and for the clear gelatin-based phantom, ultrasound was not needed as the needle was well visualized throughout the insertion process. The IMD was then deployed by advancing it forward out of the needle lumen using an inner stylet. The needle was removed, leaving the IMD embedded within the tissue and the attached guidewire externalized. The IMDs were left in place for 24 h prior to additional force testing and retrieval, simulating current pre-clinical and clinical trial workflow for the drug-screening IMD. 

### 2.3. IMD Dislodgement Force Testing

#### 2.3.1. Experimental Set-Up

**Figure 3:**
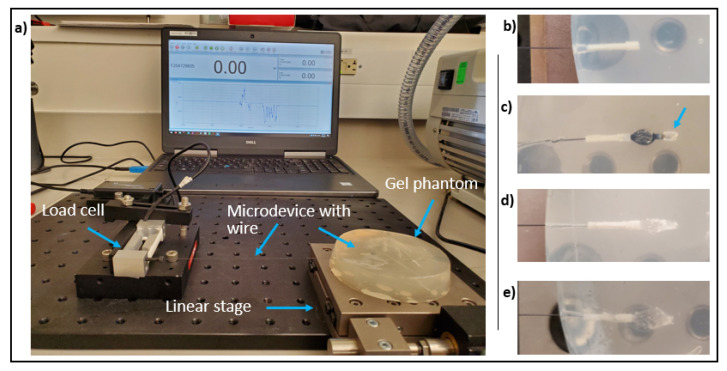
(**a**) Experimental set-up for microdevice stability force testing. A load cell is used to measure the tensile force required to displace a microdevice in a phantom model. Non-anchored (**b**), nitinol-anchored (**c**), low-absorption hydrogel-anchored (**d**), and high-absorption hydrogel-anchored (**e**) microdevices are well seen in the phantom. Microdevice displacement is also easily detected under direct visualization (blue arrow in **c**). The force at which displacement/dislodgement is first visualized is recorded and compared between each anchoring method.

**Figure 4:**
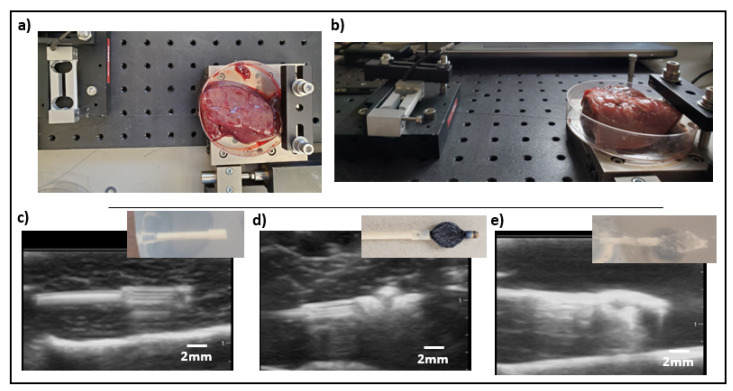
Representative force testing set-up in liver (**a**) and kidney (**b**) samples. Non-anchored (**c**), nitinol-anchored (**d**), and hydrogel-anchored (**e**) microdevices are well seen by ultrasound in all tissues at the implanted depths. Insets show the actual appearance of each device corresponding to its ultrasound image. Ultrasound was used to monitor for microdevice dislodgement in all solid tissues.

For each IMD (non-anchored, nitinol-anchored, and hydrogel-anchored), we quantified the resistance to dislodgement by applying a tensile force on the IMD (Figure 3). We have previously observed migration predominantly along the long axis of the cylindrical IMD. Therefore, resistance to tensile force along this axis was felt to be the strongest quantifiable predictor of stability for our application. The externalized nitinol wire connected to the IMD was secured to a 300 g capacity, 0.06 g accuracy resistive load cell (Loadstar Sensors). The wire was slowly pulled in 1 um increments using a linear stage (LNR502, Thorlabs, Inc., Newton, NJ, USA) and the resulting tensile force was monitored in real time using commercial software (LoadVUE LV-1000, Loadstar Inc., Fremont, CA, USA). The IMD position was continuously monitored using direct visualization in the clear phantom (Figure 3b–e), and using ultrasound (Butterfly iQ, Butterfly Networks, Inc., Guilford, CT, USA) for the opaque tissue models (Figure 4). The maximum force required to displace/dislodge the IMD from the surrounding tissue was recorded. 

#### 2.3.2. Statistical Analysis

The force testing was repeated with five replicates in each phantom and tissue, for each IMD type. Mean and standard deviation maximal dislodgement forces were calculated, and analysis of variance (ANOVA) tests were used to compare the forces required between each anchoring method and among the tested tissues. MATLAB software with statistical analysis package (R2020a, Mathworks) was used for all statistical analyses.

### 2.4. IMD Retrieval

We have previously developed a method for minimally invasive removal of implanted IMDs [14]. The method involves passage of a retrieval tool over a guidewire to the edge of the IMD. Subsequently a cutting needle similar to a biopsy needle cuts around the IMD, separating it from the adjacent tissue. This allows the IMD to be removed intact in its entirety without requiring a more invasive and higher-risk surgical approach. An ideal anchoring approach would be compatible with this method, thus preserving the ability of the IMD to be removed in a minimally invasive manner. Using our IMD retrieval tool, we performed minimally invasive retrieval of each anchored microdevice (Supplementary Figure S1c–f). Successful retrieval was defined as removal of the entire microdevice assembly without fracture or anchor dislodgement, or inadvertent retention of any IMD components in the tissue. Maximum tensile force on the IMD during retrieval was also recorded. 

## 3. Results

### 3.1. Minimally Invasive Implantation and Retrieval

Each version of the IMD (non-anchored, nitinol-mesh-anchored, and hydrogel-expansion-anchored) were deployed into the phantom and ex vivo tissues via a 17-gauge needle. After deployment, the nitinol mesh expanded to a maximal diameter of 3.2 ± 0.03 mm in phantom. Maximum expansion occurred immediately after deployment. The hydrogel anchor expanded to a maximum diameter of 2.7 ± 0.2 mm and 3.4 ± 0.3 mm for the low-absorption and high-absorption models, respectively. Maximal expansion occurred by approximately 6 h after implantation for the hydrogel anchors. 

The nitinol-based self-expansion occurred at the distal end of the IMD, away from the drug release sites. This preserved the IMD’s drug-screening functionality. For the hydrogel anchors, a similar spatially constrained self-expansion was achieved by loading the hydrogel particles within a mesh netting attached to one end of the microdevice. This ensured that the hydrogel expansion was limited to the distal end of the IMD, away from the drug release sites, thus also preserving its functionality.

Both anchored IMDs were able to be retrieved intact in a minimally invasive manner, using our 14-gauge retrieval device, without damage or retention of any part of the anchor or IMD. The coring needle enclosed and partially re-collapsed the nitinol and hydrogel anchors, thus completely separating the IMD from the surrounding tissues and facilitating its removal. Therefore, minimally invasive implantation and retrieval of an IMD for drug response evaluation was possible with both anchoring methods.

### 3.2. IMD Dislodgement Force Testing

**Figure 5:**
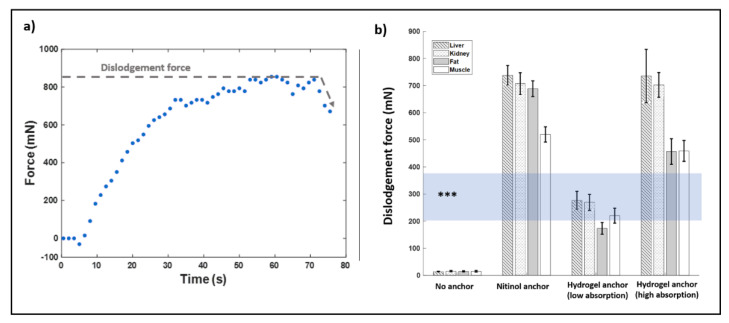
(**a**) Example force vs. time curve, obtained from nitinol-anchored microdevice in phantom model (mN = millinewtons, s = seconds). Microdevice dislodgement typically correlated with a sharp decrease in measured force (dotted line). The maximum force prior to this decline was recorded as the dislodgement force. (**b**) Dislodgement forces compared among anchoring methods and tissue types. The shaded horizontal region (***) corresponds to force ranges required for minimally invasive retrieval of the IMDs, as previously described [14].

A characteristic force vs. time curve from the dislodgement testing is shown in Figure 5a. For the anchored devices, we observed an initial gradual increase in tensile force. This corresponded to stable positioning without dislodgement, as observed by direct visualization in the phantom samples and by ultrasound in the tissue samples. This was followed by a steep drop in tensile force, which corresponded visually to IMD dislodgement and migration within tissues. 

Summary performance data for each anchoring method in each tissue type is presented in Figure 5b and Table 1. Both the nitinol-anchored IMD and hydrogel-anchored IMDs required significantly greater force for dislodgement in all tested media compared with non-anchored devices (*p* < 0.001 for all pairwise comparisons). The high-absorption hydrogel-anchored IMDs and nitinol-anchored IMDs had the best overall performance. The nitinol anchor performed comparatively better in fat tissues (*p* < 0.001), but the two methods were not significantly different in any of the other tissues, at *p* < 0.05 significance level. Both of these methods were significantly better compared to the low-absorption hydrogel-anchored IMDs in all tissues (*p* < 0.01 for all pairwise comparisons). The hydrogel anchors provided greater stability to dislodgement in liver and kidney tissues compared to fat and muscle tissues (*p* < 0.05 for each pairwise comparison). The nitinol anchors provided greater stability to liver, kidney, and fat tissues compared to muscle (*p* < 0.05).

The horizontal semi-transparent zone in Figure 5b (marked by asterisks: “***”) represents tensile forces on our drug-screening IMD during minimally invasive retrieval, which roughly estimates maximum in vivo forces based on prior experience in murine subcutaneous flank tumor models [14]. The non-anchored IMDs dislodged at forces below those observed during retrieval, suggesting that the non-anchored approach may not be feasible for this application. The hydrogel-anchored devices with small particles provided resistance to dislodgement at the lower limit of retrieval forces but not the upper limit, and therefore may fail in some IMD settings. Finally, the nitinol-anchored and hydrogel-anchored IMDs with large particles provided resistance to dislodgement at forces greater than all observed forces during retrieval, suggesting that these may be effective in most settings for our IMD application.

## 4. Discussion

This is the first study to quantify mechanical stabilizing effects of novel hydrogel and nitinol self-expanding anchors across a range of biological tissues. Our results overall suggest that both anchoring methods could be incorporated into implanted microdevices to maximize their stability and efficacy. They are both compatible with a minimally invasive approach, which should increase the overall safety and practicality of use in a variety of clinical settings.

Both microdevices anchored with the nitinol and high-absorption hydrogel self-expansion performed better than non-anchored control devices in all tissues and increased overall stability of the microdevices by a factor of 30–50×. The low-absorption hydrogel-anchors did not perform as well, likely because they had more limited expansion after deployment. This indicates that the degree of stability is related to the extent of expansion and contact surface area after deployment. This dependence provides a potential mechanism that can be used to customize the anchor to specific tissues, and to provide greater resistance to migration by increasing the maximum hydrogel expansion. However, anchor expansion would have to be tailored to the specific clinical setting and anatomic location, as it would increase the overall footprint of the microdevice and could make retrieval more challenging.

The hydrogel anchors were found to be more effective in liver and kidney tissues compared to fat and muscle. One possible explanation for this is that liver and kidney tissues have relatively higher water content [23], which may promote hydrogel expansion. Another possible explanation is that these tissues are composed of tightly packed organized functional units (e.g., hepatic lobules in liver and nephrons in the kidney) and are more dense than fat and muscle [24], which may also contribute to microdevice stability. The nitinol anchors, although overall effective in all tissues, provided relatively less resistance to dislodgment in muscle. One possible explanation is that the striated skeletal muscle fibers are organized in linear bands that are not as tightly packed as the more homogeneous and uniform fat, liver, and kidney tissues, and therefore may be more susceptible to microdevice migration.

We have specifically tested performance after integration with our drug-screening IMDs; however, the anchors used here can be readily integrated into a wide range of implantable technologies using a similar approach. For example, brachytherapy seed and fiducial markers are used for delivering and guiding radiation treatment of cancers in a variety of locations including the liver, subcutaneous and retroperitoneal fat, prostate, and musculoskeletal tumors. Migration of these devices is not uncommon and can cause clinically adverse outcomes, as previously described [15,16,25]. Other preclinical implantable microdevices, including those recently developed for early disease detection and controlled drug delivery [4,5], also require precise placement and stability, and would likely benefit from the anchoring methods described here. Our quantitative data support the use of both nitinol and hydrogel self-expanding anchors to minimize migration risk.

Although our results in ex vivo tissues are promising, the anchored microdevices will have to be tested in animal and ultimately human trials to confirm feasibility for specific in vivo applications. In vivo forces on implanted microdevices are multifactorial and include anatomic site, physiologic tissue movement (e.g., breathing), patient activity, body habitus, tissue laxity, intrinsic tissue contraction and expansion, and numerous other factors [26]. Given the complexity of these internal tissue biomechanics and significant variability among different patients and tissue types, it is not possible to generalize the forces that act on implanted microdevices in all clinical settings. Therefore, dislodgement force testing as assessed in this study is the most direct and quantitative measure of anchor performance. However, in vivo testing in animal and human studies in a variety of settings will be important to fully assess and realize the potential of these anchoring methods. Preclinical studies evaluating stability of our specific drug-screening IMDs in various anatomic sites are planned.

It is worth noting that the current study only evaluated resistance to tensile force along the long axis of our cylindrical microdevice. Although rotational movement and lateral migration perpendicular to the microdevice long axis are theoretically possible, we have not observed this to any significant extent in our prior studies in pre-clinical and early clinical trials. Finally, biological tissues are known to grow around and adhere to implanted materials, and this effect increases with duration of implantation and contact surface area [27]. Therefore, by the same mechanism that they provide mechanical stability, the anchors will also likely make microdevice retrieval more difficult, particularly with longer term implantations of several weeks to months. While we have shown that our microdevices can be removed percutaneously using our custom retrieval needle, the theoretical risk of bleeding and/or tissue injury associated with anchored microdevice removal in an in vivo setting may be increased, and will require additional pre-clinical testing prior to clinical implementation.

## 5. Conclusions

In conclusion, we have demonstrated the use of nitinol- and hydrogel-based anchors to stabilize percutaneously implanted microdevices in a variety of solid tissues, and have shown that they significantly improve microdevice stability by a factor of 30–50× compared with non-anchored microdevices. Our results strongly support further utilization of these anchoring methods, which can be readily integrated into a variety of existing and emerging microdevice technologies to improve their efficacy and reduce migration-induced complications.

## Figures and Tables

**Table 1:** Maximum forces required to dislodge IMDs based on tissue type and anchoring method. Mean and standard error of mean (SEM) values are presented.

	Dislodgement Force, milliNewtons (mN)				
	Phantom	Liver	Kidney	Fat	Muscle
Non-anchored	30.4 ± 4.9	13.3 ± 1.6	15.2 ± 2.6	14.8 ± 2.0	15.0 ± 2.9
Nitinol-expansion-anchored	887.3 ± 20.7	737.5 ± 36.5	707.1 ± 40.3	688.3 ± 29.0	519.8 ± 28.1
Hydrogel-anchored (low absorption)	472.9 ± 34.3	276.3 ± 33.4	269.2 ± 29.4	173.3 ± 21.7	220.1 ± 27.6
Hydrogel-anchored (high absorption)	873.3 ± 43.9	735.2 ± 98.2	702.4 ± 45.5	456.6 ± 47.3	458.9 ± 38.5

## Data Availability

The data presented in this study are openly available in Harvard. Dataverse repository at https://doi.org/10.7910/DVN/GRQDDO.

## Supplementary Materials

The following are available online at https://www.mdpi.com/article/10.3390/mi12040404/s1, Figure S1. (a–b) Minimally invasive implantation of microdevice using a 17-gauge needle. Needle preloaded with the microdevice and collapsed anchor is passed into a phantom (a), and the microdevice is pushed out using an inner stylet (b), immediately allowing the anchor to expand. (c–f) A custom retrieval device (detailed in ref [14]) is used to percutaneously remove the microdevice from the phantom. The retrieval needle is passed over the guidewire (c) to the edge of the microdevice (d). A coring needle then passes around the microdevice to sever it from the surrounding tissue (e) and the microdevice is pulled out of the phantom in its entirety (f). This allows the microdevice to be implanted and removed without requiring a more invasive and high-risk surgical approach.
