# Supplementary material for: Self-Expanding Anchors for Stabilizing Percutaneously Implanted Microdevices in Biological Tissues
Source: Micromachines (Basel). 2021 Apr 6;12(4):404. doi: 10.3390/mi12040404 (PMC8067345; doi:10.3390/mi12040404)
Supplement: Supplementary file 1 [file micromachines-12-00404-s001.pdf]

micromachines

Supplementary Materials

Self-Expanding Anchors for Stabilizing Percutaneously Implanted Microdevices in Biological Tissues

Sharath Bhagavatula *, Devon Thompson, Christine Dominas, Irfanullah Haider and Oliver Jonas *

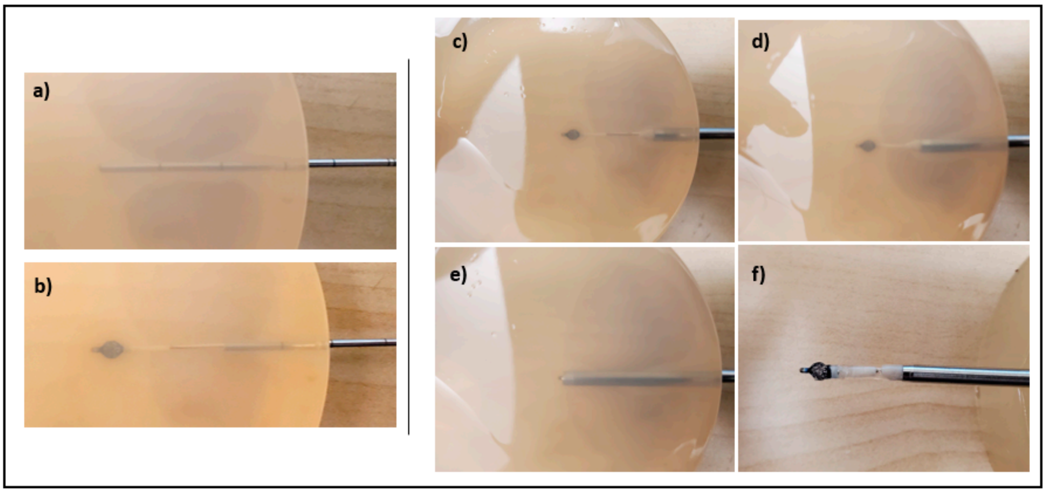

Figure S1. (**a**–**b**) Minimally invasive implantation of microdevice using a 17-gauge needle. Needle preloaded with the microdevice and collapsed anchor is passed into a phantom (**a**), and the microdevice is pushed out using an inner stylet (**b**), immediately allowing the anchor to expand. (**c**–**f**) A custom retrieval device is used to percutaneously remove the microdevice from the phantom. The retrieval needle is passed over the guidewire (**c**) to the edge of the microdevice (**d**). A coring needle then passes around the microdevice to sever it from the surrounding tissue (**e**) and the microdevice is pulled out of the phantom in its entirety (**f**). This allows the microdevice to be implanted and removed without requiring a more invasive and high-risk surgical approach.

Citation: Bhagavatula, S.; Thompson, D.; Dominas, C.; Haider, I.; Jonas, O. Self-Expanding Anchors for Stabilizing Percutaneously Implanted Microdevices in Biological Tissues. *Micromachines* **2021**, *12*, 404. https://doi.org/10.3390/mi12040404

Academic Editor: Gerard Cummins
Received: 12 March 2021
Accepted: 1 April 2021
Published: date

Publisher's Note: MDPI stays neutral with regard to jurisdictional claims in published maps and institutional affiliations.

Copyright: © 2021 by the authors. Licensee MDPI, Basel, Switzerland. This article is an open access article distributed under the terms and conditions of the Creative Commons Attribution (CC BY) license (http://creativecommons.org/licenses/by/4.0/).
